# Carrier Modulation via Tunnel Oxide Passivating at Buried Perovskite Interface for Stable Carbon-Based Solar Cells

**DOI:** 10.3390/nano13192640

**Published:** 2023-09-26

**Authors:** Yuqing Xiao, Huijie Zhang, Yue Zhao, Pei Liu, Kiran Kumar Kondamareddy, Changlei Wang

**Affiliations:** 1 School of Automation, Zhongkai University of Agriculture and Engineering, Guangzhou 510225, China; 2 Key Laboratory of Artificial Micro & Nano Structures of Ministry of Education, School of Physics and Technology, Wuhan University, Wuhan 430072, China; 3 Key Laboratory of Advanced Optical Manufacturing Technologies of Jiangsu Province & Key Laboratory of Modern Optical Technologies of Education Ministry of China, School of Optoelectronic Science and Engineering & Collaborative Innovation Center of Suzhou Nano Science and Technology, Soochow University, Suzhou 215006, China; 4 Department of Physics, School of Pure Sciences, College of Engineering Science and Technology, FIJI National University, Lautoka Campus, Suva 744101, Fiji

**Keywords:** perovskite solar cells, insulating layer, carrier modulation, carbon electrodes

## Abstract

Carbon-based perovskite solar cells (C-PSCs) have the impressive characteristics of good stability and potential commercialization. The insulating layers play crucial roles in charge modulation at the buried perovskite interface in mesoporous C-PSCs. In this work, the effects of three different tunnel oxide layers on the performance of air-processed C-PSCs are scrutinized to unveil the passivating quality. Devices with ZrO_2_-passivated TiO_2_ electron contacts exhibit higher power conversion efficiencies (PCEs) than their Al_2_O_3_ and SiO_2_ counterparts. The porous feature and robust chemical properties of ZrO_2_ ensure the high quality of the perovskite absorber, thus ensuring the high repeatability of our devices. An efficiency level of 14.96% puts our device among the state-of-the-art hole-conductor-free C-PSCs, and our unencapsulated device maintains 88.9% of its initial performance after 11,520 h (480 days) of ambient storage. These results demonstrate that the function of tunnel oxides at the perovskite/electron contact interface is important to manipulate the charge transfer dynamics that critically affect the performance and stability of C-PSCs.

## 1. Introduction

Organic lead halide perovskite solar cells (PSCs) have emerged as a competitor of silicon photovoltaics regarding their high performance and commercial prospects. During the last few years, the power conversion efficiency (PCE) of PSCs improved from 3.8% to a recently certified 26.1% [1,2] as a result of relying on perovskite films with impressive properties, such as a high absorption coefficient, excellent ambipolar charge transport [3,4,5], long carrier diffusion lengths and a tunable bandgap [6,7,8,9,10]. Noble metals and organic hole-transporting materials (HTMs) are being widely employed for the preparation of state-of-the-art PSCs. However, their presence leads to an expensive manufacturing process and poor stability [11,12,13,14]. These issues could be overcome by the application of carbon counter electrodes (CEs) in hole-conductor-free PSCs. However, the removal of hole collection layers would sacrifice cell efficiency [15,16,17,18,19]. Currently, the PCE of hole-conductor-free C-PSCs is still lower than 20%, considerably lagging behind regular PSCs with fully functional layers [20,21].

Electron-collecting contacts play vital roles in determining the performance of common PSCs [22,23,24,25,26,27], especially that of carbon-based hole-transporting-layer-free PSCs (C-PSCs) [28,29,30]. Electron contacts simultaneously affect the charge transfer dynamics and influence the growth kinetics of the perovskite absorber [31]. Despite the fast progress and superior stability of C-PSCs [32], more in-depth research efforts are still required to improve the performance of C-PSCs. Since there are no HTMs in C-PSCs, charge manipulation and film growth modulation are more important than PSCs with fully functional layers [33]. Spike energy strategies that reduce the interface recombination are highly desired in inorganic photovoltaics [34,35]. SiO_2_ and Al_2_O_3_ have been introduced in silicon photovoltaics as electron tunneling paths forming the tunnel oxide passivated contacts on solar cells [36]. Similarly, insulating layers acting as energy band uplifters at the perovskite/TiO_2_ electron transport layer (ETL) interface are usually employed in mesoscopic PSCs [37,38,39]. Han and coworkers reported that modifying the ETL surface with an insulating material reduces the charge recombination and improves the open-circuit voltage (V_oc_) of the device PSCs [37]. Xu and coworkers found that introducing a thick (about 100 nanometers) Al_2_O_3_ insulator layer can reduce nonradiative recombination in PSCs [40]. Kamat and coworkers reported that hole accumulation can indirectly promote halide ion segregation in HTM-free PSCs with TiO_2_ ETLs, while insulating ZrO_2_ substrates suppresses phase segregation due to a more balanced charge transport [41].

In addition, the surface morphology of the underneath scaffold has a strong impact on the perovskite layer, which is paramount in influencing the final efficiency of PSCs. Zhu and coworkers investigated the compositional and optoelectronic properties of the buried perovskite interface [42]; they found that the bottom surfaces of perovskite films have severe compositional inhomogeneity and sub-microscale imperfections, causing major energy loss pathways that hinder device performance. They suggest that the underneath scaffolds play vital roles in the elimination of detrimental defects on the perovskite bottom surfaces. Therefore, surface topography tailoring should also be significantly considered in the optimization of C-PSCs. Regarding the charge transfer dynamics and perovskite film crystallization kinetics, the application criteria of Al_2_O_3_ [37], ZrO_2_ [18,38] and SiO_2_ [39] should be unveiled urgently.

In this work, we investigated the influence of tunnel oxide passivating (TOP) layers on the perovskite film quality and charge transporting properties of mesoscopic C-PSCs. TOP layers have several advantages in C-PSCs: first, they uplift the band bending at perovskite/ETL interfaces through the passivation of TiO_2_ surfaces by forming a discontinuous coating; second, they reduce charge shunting risks in the case of the presence of pinholes in the perovskite film; and, third, they modify the ETL with a porous topology more favorable for the solution infiltration of the perovskite precursor, leading to a higher absorber film quality and better interconnection with ETL networks. We selected commonly employed dielectric materials as tunnel oxides in PSCs, including Al_2_O_3_, SiO_2_ and ZrO_2_. In particular, ZrO_2_ has a relatively higher dielectric constant than TiO_2_, which might cause sufficient passivation on the TiO_2_ surface. Moreover, ZrO_2_ nanoparticle-coated scaffolds maintain high uniformity and porous features, facilitating perovskite crystallization and charge collection. An electrical impedance spectroscopy demonstrated that ZrO_2_ TOP-based C-PSCs show the best charge transfer properties with the highest efficiency of 14.96%. The efficient passivation with the tunnel oxide layer enables the high repeatability of our devices. Our HTM-free C-PSCs were fabricated under ambient conditions with a humidity of about 50%, further emphasizing the robust air stability that is compatible with high-yield manufacturing processes. Our C-PSCs present excellent long-term stability; they maintained 88.9% of their original efficiency after 11,520 h (480 days) of ambient storage without encapsulation.

## 2. Materials and Methods

### 2.1. Materials

Lead iodide (PbI_2_, 99.99%), CH_3_NH_3_I (MAI), bis(2,4-pentanedionato)-bis(2-propanolato)titanium(IV) (C_16_H_28_O_6_Ti) (75wt% in isopropanol) were purchased from TCI, isopropanol (IPA, 99.8%), diethanolamine (DMF, 99.8%), lead chloride (PbCl_2_, 99.99%), 1-butanol (99.8%), ZrO_2_ (50 nm, 99.99%), SiO_2_ (50 nm, 99.5%) and Al_2_O_3_ (γ phase, 20 nm, 99.99%)nanoparticles were obtained from Aladdin. The conductive carbon paste was synthesized according to our previous work [43].

### 2.2. Preparation of TiO_2_, ZrO_2_, Al_2_O_3_, SiO_2_ Paste

ZrO_2_, Al_2_O_3_ andSiO_2_ pastes were prepared by ball-milling 1.4 g powders of commercial ZrO_2_, Al_2_O_3_, SiO_2_, 0.7 g ethylcellulose, 5.77 g terpineol and 23.61 g ethanol for 24 h. The as-prepared ZrO_2_ and Al_2_O_3_ pastes were subsequently diluted with ethanol in ratios of 1, 2, 3 and 4 times by weight to obtain the optimal thickness of tunnel oxide layers. The high concentration of original pastes resulted in excessively thick insulating layers on the surface of the TiO_2_ electron transporting layer (ETL), thereby impeding charge transport and reducing the performance of C-PSCs. The TiO_2_ paste was prepared using a recipe that was similar to the published work [44].

### 2.3. Fabrication of C-PSCs

The process for fabricating C-PSCs involved several steps. First, the FTO glass was patterned by etching with Zn powder and 2 M HCl diluted in ethanol. The surface of the glass was then cleaned using acetone, deionized water, acetone and ethanol alternately, and dried in clean air. A solution of 0.15 M titanium diisopropoxidebis(acetylacetone) in 1-butanol was spin-coated on the cleaned FTO glass at 3000 rpm for 30 s, and dried at 125 °C for 20 min to form a compact TiO_2_ layer. The mesoporous TiO_2_ layer was deposited over the compact TiO_2_ layer by spin-coating a homemade TiO_2_ P25 paste at 3000 rpm for 20 s. The deposited layers were then sintered in air at 500 °C for 30 min. After cooling down to room temperature, the films were treated with a 0.05 M aqueous solution of TiCl_4_ at 70 °C for 30 min, rinsed with deionized water and ethanol, and dried in the air. Insulating layers were prepared by spin-coating ZrO_2_, Al_2_O_3_ or SiO_2_ paste over the TiCl_4_-treated TiO_2_ film and annealing it at 500 °C for 30 min. The perovskite film was deposited on the mesoporous TiO_2_ film using a two-step sequential method under ambient conditions with high humidity (~50%). In the first step, the PbI_2_ precursor solution was spin coated at 4000 rpm for 20 s and then the wet PbI_2_ film was treated with ethanol and annealed at 100 °C for 8 min. In the second step, the film was immersed in an isopropanol solution of MAI (7 mg mL^−1^) for 5 min and dried with nitrogen gas. The as-prepared MAPbI_3_ perovskite film was further heated at 100 °C for 10 min. Finally, the carbon paste was coated over the perovskite film using a doctor-blade method and annealed at 100 °C for 40 min. The resulting solar cells had a configuration of FTO/c-TiO_2_/meso-TiO_2_/TOP layer/MAPbI_3_/carbon.

### 2.4. Measurements and Characterization

The Bruker instrument (D8 Focus diffractometer) utilizing Cu Kα radiation (λ = 0.15406 nm) at 40 kV and 40 mA was employed for structural analysis. The surface and cross-sectional morphology were observed by a field emission scanning electron microscope (SEM, Zeiss SIGMA, Jena, Germany). The absorption spectra of films deposited on FTO were collected using a UV-vis spectrophotometer (Lambda 650S, PerkinElmer, Shelton, CT, USA) with a wavelength range of 300–800 nm at room temperature. The J-V curves of the solar cells were measured using a CHI660C electrochemical workstation (Shanghai, Chenhua) coupled with a solar simulator (Newport, 91192) under 100 mW cm^−2^ illumination (AM 1.5 G) with a scan rate of 0.05 V s^−1^. The area of the portion of C-PSC exposed to the radiation was confined to 0.1 cm^2^ using a metal mask. The films were characterized by ambient air condition, with a temperature of around 25 °C and relative humidity of 50%. The time-resolved photoluminescence (TRPL) was performed using a time-correlated single photon counting (TCSPC) module, excited with a 532 nm pulsed laser. The external quantum efficiency (EQE) was measured using an instrument equipped with a 300 W xenon lamp (Newport 66984), and the monochromatic light ranged from 300 to 800 nm. Electrochemical impedance spectra (EIS) were recorded under one sun illumination over the range of frequencies spanning from 1 MHz to 1 Hz at open-circuit voltage bias. During the long-term stability test, we stored the devices under room light without further protection. As aging progressed, the devices exhibited gradually increased performance for hundreds of hours before beginning to decline. The devices were exposed to room light during storage. 

## 3. Results and Discussion

**Figure 1:**
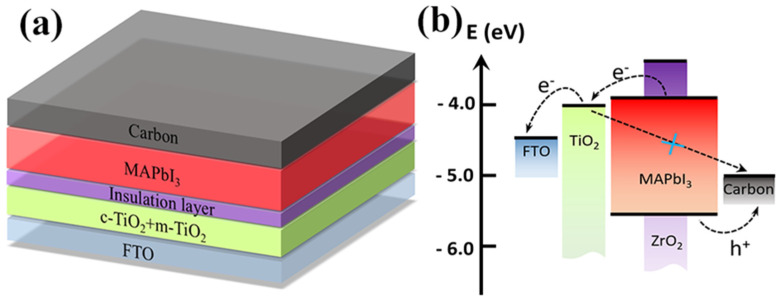
(**a**) Schematic illustration of the C−PSCs structure; (**b**) energy−level diagram of a C−PSC with ZrO_2_ as a TOP layer.

The C-PSCs possess a straightforward device architecture comprising FTO/TiO_2_/TOP layer/perovskite/carbon, as depicted in Figure 1a. Here, FTO refers to fluorine-doped tin oxide, and the perovskite layer corresponds to CH_3_NH_3_PbI_3_ (MAPbI_3_). The entire fabrication process was conducted in ambient air, with the perovskite layer deposited using a two-step sequential method, and the carbon electrode doctor-blade coated onto the perovskite film using the homemade carbon paste [43]. Our previous research indicates that high-temperature annealed TiO_2_ films exhibit numerous surface defects, which should be responsible for the inferior performance of C-PSCs [44]. We randomly used TiCl_4_ post-treatment and an external SiO_2_ coating to passivate the ETL surfaces, and the PCEs have been improved for the corresponding C-PSCs due to the elimination of interface defects [38,44]. However, the criteria for selecting surface passivating layers remain unclear. Drawing inspiration from the optimization processes of silicon (Si) solar cells, we deliberately selected SiO_2_, Al_2_O_3_ and ZrO_2_ as the TOP layers, taking into account their surface charge states, dialectical constants, film topologies and interface electric fields. Efficient C-PSCs require optimal TOP layers. Thus, we initially investigated the concentrations of SiO_2_, ZrO_2_ and Al_2_O_3_ pastes by diluting the original pastes with ethanol. Figure 1b shows the typical energy level diagram of the C-PSC that employs the ZrO_2_ TOP layer. The thin ZrO_2_ TOP layer exhibits a deep valence band maximum (VBM), with its conduction band position surpassing that of TiO_2_ [37]. Photo-generated electrons in the perovskite absorber layer may transfer from the conduction band (CB) of MAPbI_3_ to TiO_2_ through either the tunneling effect or the voids of the discontinued insulating layer coated on the thick mesoporous TiO_2_ scaffold. Consequently, electrons accumulate at the TiO_2_ interface due to the existence of this layer of insulating oxide, ultimately elevating the Fermi level and increasing the V_oc_ of the solar cell. Simultaneously, due to the blocking effect of the insulating layers, it becomes challenging for electrons in the conduction band of TiO_2_ to recombine with holes. Therefore, a thin layer of insulating materials can reduce interfacial recombination, thereby facilitating carrier transport [40].

**Figure 2:**
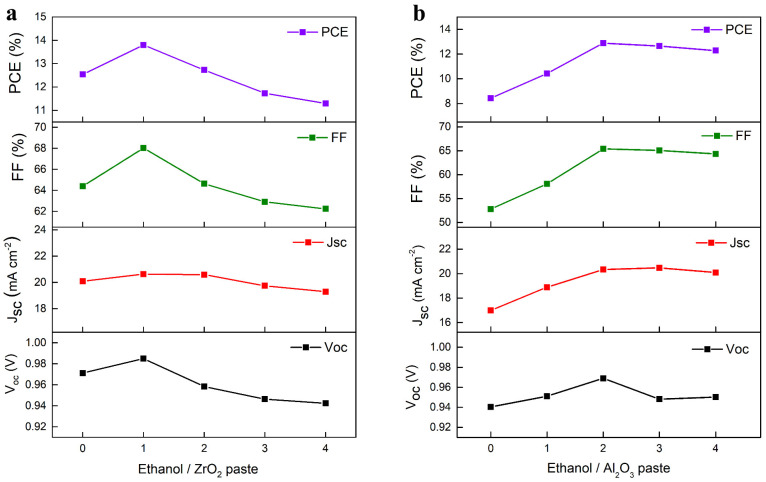
Dependence of V_oc_, J_sc_, FF and PCE on the concentration of (**a**) ZrO_2_ paste and (**b**) Al_2_O_3_ paste.

The variation in photovoltaic parameters of C-PSCs employing different concentrations of ZrO_2_ and Al_2_O_3_ pastes are shown in Figure 2a,b, respectively. As the concentration of ZrO_2_ or Al_2_O_3_ increases, the photovoltaic parameters, including V_oc_, J_sc_, FF and PCE, first exhibit an increase and then a decrease trend. This can be ascribed to the thickness of the tunnel oxide layers, which is tuned by the concentration of the pastes. For the non-treated C-PSCs, severe charge recombination occurs at the buried perovskite interface due to the presence of defects, leading to inferior performance. However, if the concentration of ZrO_2_ or Al_2_O_3_ is too high, the thickness will be thick enough to suppress the electron transportation process, ultimately reducing the performance of final devices. The optimal weight ratio of ethanol to ZrO_2_ and Al_2_O_3_ pastes is found to be 1:1 and 2:1, respectively. The SiO_2_ TOP layer used here is the same as that reported in our previous work [38].

**Figure 3:**
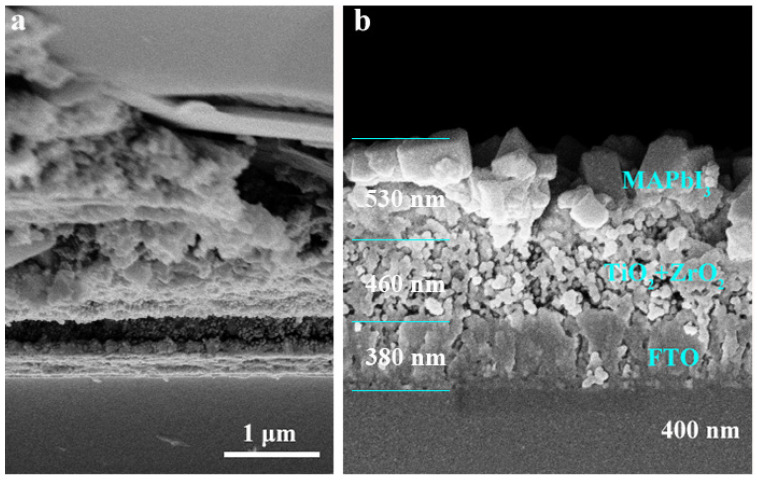
(**a**) Cross-sectional SEM image of the PSC device; (**b**) cross-section SEM image of perovskite grown on TiO_2_/ZrO_2_ layer.

Figure 3a,b shows the cross-sectional SEM images of the full device and perovskite film grown on the TiO_2_/ZrO_2_ layer, respectively. The thicknesses of FTO, TiO_2_/ZrO_2_ and MAPbI_3_ are about 380 nm, 460 nm and 530 nm, respectively. The images reveal that the perovskite materials infiltrate well into the pores and the carbon electrode tightly adheres to the perovskite film.

**Figure 4:**
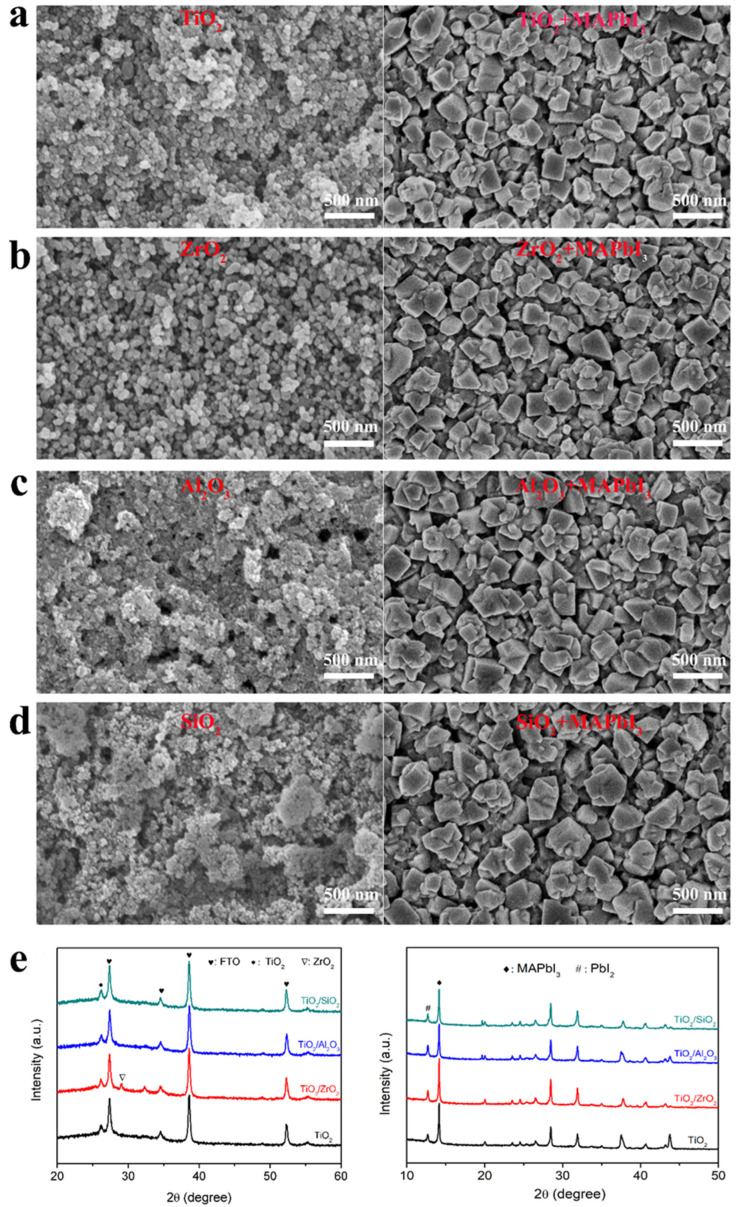
(**a**–**d**) Plane-view SEM images of TiO_2_ film, TiO_2_/ZrO_2_ film, TiO_2_/Al_2_O_3_ film and TiO_2_/SiO_2_ film (left column) and the corresponding perovskites grown on them (right column); (**e**) XRD patterns of TiO_2_, TiO_2_/ZrO_2_, TiO_2_/Al_2_O_3_ and TiO_2_/SiO_2_ films (**left**) and the corresponding perovskite films (**right**) coated on them.

The surface morphology and structure of scaffold layers play a crucial role in the performance of mesoscopic PSCs. Factors such as surface roughness, pore size and hydrophilicity have a significant impact on the infiltration of perovskite materials, crystallization quality and carrier transportation in the device [37]. Figure 4a–d shows the surface morphology of various scaffold layers (left column) and the perovskite grown on them (right column). TiO_2_ film shows a relatively uniform surface, with some nanoparticle agglomeration. In contrast, the TiO_2_/ZrO_2_ film exhibits a homogeneous morphology with well-dispersed top ZrO_2_ nanoparticles, which facilitates the infiltration and growth of perovskite materials, thereby promoting the transport of photo-generated carriers. However, the pores in the TiO_2_/Al_2_O_3_ scaffold are very small, which will hinder the infiltration of the precursor solution and limit the growth and crystallization of MAPbI_3_ in the pores. The TiO_2_/SiO_2_ film shows serious agglomeration, resulting in a rough surface with an exposed TiO_2_ layer that weakens the effect of the insulation layer as a separator between the carbon electrode and TiO_2_ layer, leading to a higher risk of shunting. All perovskite films grown on different scaffold layers show nanocube-like structures, indicating that the addition of insulating oxide has little effect on their surface morphology. Therefore, the improvement in PSCs’ performance is not only caused by the morphology of the perovskite but also by the modulation of the TOP layer on the carriers, which will be discussed later.

Figure 4e shows the XRD patterns of TiO_2_, TiO_2_/ZrO_2_, TiO_2_/Al_2_O_3_ and TiO_2_/SiO_2_ films. Except for the TiO_2_/ZrO_2_ film that shows a ZrO_2_ tetragonal phase at 2θ~29.2° [45], the XRD patterns for the rest of the films are identical to that of the TiO_2_ film without new peaks belonging to SiO_2_ or Al_2_O_3_. This means that the SiO_2_ or Al_2_O_3_ present in the TiO_2_ film is in the amorphous phase rather than the crystal phase, which could be attributed to the higher sintering temperatures required to form the phases [46,47]. The XRD patterns of perovskite films coated on different metal oxide films reveal similar features, indicating that the introduction of insulating layers does not affect the crystallization of perovskite inside. The diffraction peak observed at around 12.7° corresponds to PbI_2_, resulting from the presence of excess lead iodide in the perovskite, which can contribute to the increase in the V_oc_ of PSCs [48].

**Figure 5:**
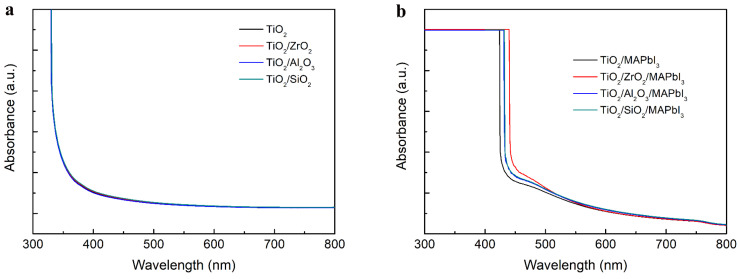
UV-vis absorption spectra of (**a**) TiO_2_, TiO_2_/ZrO_2_, TiO_2_/Al_2_O_3_ and TiO_2_/SiO_2_ films, (**b**) perovskite films coated on TiO_2_, TiO_2_/ZrO_2_, TiO_2_/Al_2_O_3_ and TiO_2_/SiO_2_.

In addition, we studied the UV-Vis absorption spectra for ETLs and MAPbI_3_ perovskite films grown over different insulating layers, as shown in Figure 5a,b, respectively. The absorption spectra of the scaffolds with various insulating layers exhibit negligible differences. The absorption of perovskite films is slightly increased after the addition of the insulating layers, which may be caused by the increased thickness of the scaffold layers resulting from the introduction of the insulating layers so that more perovskites can be loaded.

N_2_ adsorption–desorption isotherms were recorded for the powders of TiO_2_, ZrO_2_, Al_2_O_3_ and SiO_2_, as shown in Figure S1a–d. The inset shows the corresponding pore-size distribution curves obtained by the Barrett–Joyner–Halenda (BJH) method. The isotherms of the samples are the originated classic type IV isotherms of H3 hysteresis loop, indicating the existence of mesopores (2–50 nm) originating from the aggregated nanoparticles. This is consistent with the results observed from the SEM. The TiO_2_ and Al_2_O_3_ exhibit relatively narrower pore size distribution. The pore diameter of TiO_2_ ranges from 62 nm to 88 nm, while Al_2_O_3_ shows the smallest pore size of ~20 nm. The smaller pores may facilitate poor penetration of PbI_2_ into the TiO_2_ mesoporous scaffold and hinder the growth of perovskite. However, ZrO_2_ and SiO_2_ exhibit a wide range of pore-size distribution. The ZrO_2_ is mainly composed of macropores with a size of the order of 100–165 nm, and the pore size of SiO_2_ is distributed between 50 and 120 nm. Therefore, the relatively larger pore size corresponding to the ZrO_2_ can accommodate more perovskite in the scaffold layer, which, in turn, facilitates better light harvesting and higher electron collection efficiency.

**Figure 6:**
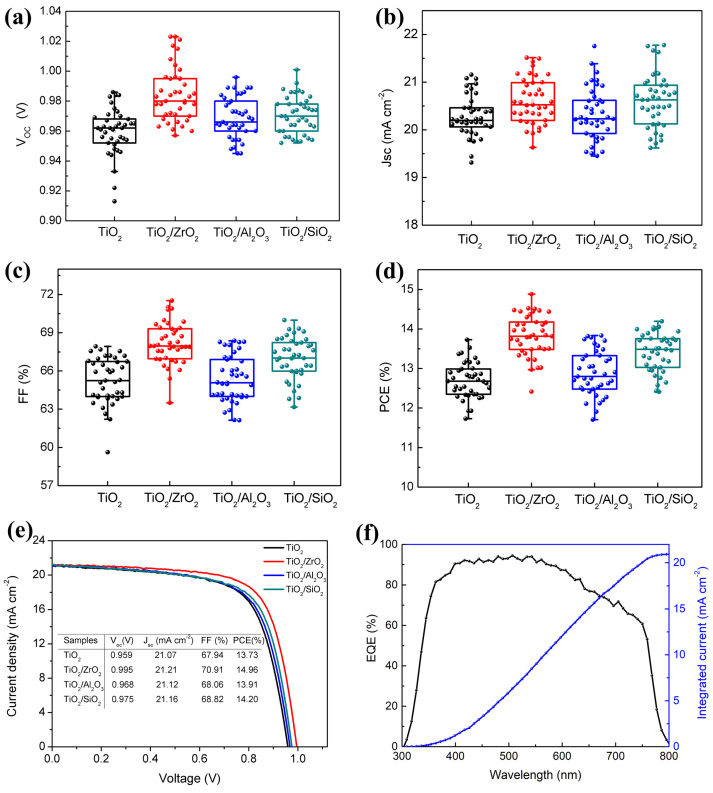
Statistics of (**a**) V_oc_, (**b**) J_sc_, (**c**) FF and (**d**) PCE for C−PSCs prepared using TiO_2_, TiO_2_/ZrO_2_, TiO_2_/Al_2_O_3_ andTiO_2_/SiO_2_ as scaffold layer (40 cells for each kind of devices); (**e**) J−V curves of the champion C−PSCs with various insulating layers; (**f**) incident photon-to-electron conversion efficiency (IPCE) spectrum and corresponding integrated current for the device based on TiO_2_/ZrO_2_.

We prepared a large number of C-PSCs to study the effects of different insulation layers on photovoltaic performance. Figure 6a–d shows the statistics of photovoltaic parameters, including V_oc_, short circuit current density (J_sc_), FF and PCE, and the corresponding average values are summarized in Table 1. Each parameter was calculated from 40 devices. The C-PSC without an insulating layer shows an average V_oc_ of 0.959 V, a J_sc_ of 20.29 mA cm^−2^ and an FF value of 65.25%, yielding an average PCE value of 12.71%. The average PCE values of C-PSCs that employ ZrO_2_, Al_2_O_3_ and SiO_2_ as insulation layers are increased to 13.84%, 12.89% and 13.42%, respectively. The increase in PCE is mainly due to the enhancement of V_oc_ and FF, which can be attributed to the inhibition of carrier recombination by insulation layers, as discussed above. As mentioned previously, after TOP layer coating, the absorption of the perovskite film slightly increases. Therefore, more light energy can be collected and the J_sc_ of the solar cell is increased accordingly. The improvement in average PCE for PSCs employing ZrO_2_ as the insulating layer is noticeably higher than that of PSCs using Al_2_O_3_ and SiO_2_. This could be due to the uniform and porous morphology of ZrO_2_ promoting the effective permeation of PbI_2_ into the mesoporous TiO_2_ scaffold layer, thereby facilitating charge transport. As shown in Figure S2, our devices exhibit good reproducibility with a small deviation in PCE.

Figure 6e shows the J-V curves of the best-performing devices with different scaffold layers, with the corresponding photovoltaic parameters listed in the inset. The device with a ZrO_2_ insulating layer exhibits excellent performance, with a V_oc_ of 0.995 V, a J_sc_ of 21.21 mA cm^−2^ and an FF of 70.91%, yielding a PCE of 14.96%. Figure 6f shows the incident photon-to-electron conversion efficiency (IPCE) spectrum of a C-PSC prepared on TiO_2_/ZrO_2_ film. The measured integral J_sc_ from the IPCE spectrum is also shown in Figure 6f. The resulting integrated J_sc_ value is 20.92 mA cm^−2^, which is only ~1.4% lower than that of the champion cell (21.21 mA cm^−2^) in Figure 6e.

**Figure 7:**
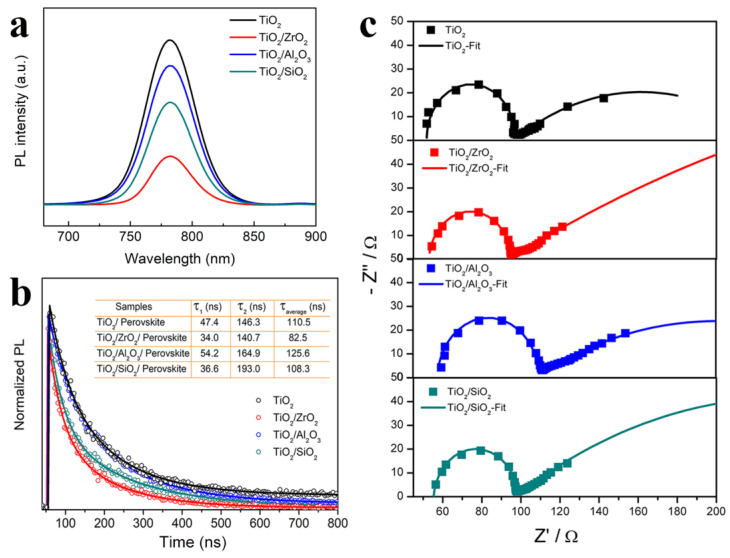
(**a**) Steady-state photoluminescence (PL) spectra and (**b**) time-resolved photoluminescence (TRPL) spectra of MAPbI_3_ films deposited on various scaffolds; (**c**) EIS spectra and their fitting curves of C−PSCs based on various insulating layers.

The perovskite films deposited on the surface of the insulating layers, as shown in Figure 7a, have a stable PL intensity lower than that deposited on the TiO_2_ surface, suggesting that the introduction of the TOP layer increases the transport and extraction efficiency of the carrier. The strongest PL quenching occurred on the perovskite film deposited on TiO_2_/ZrO_2_, indicating that ZrO_2_ has better charge modulation capabilities. We further performed a time-resolved photoluminescence (TRPL) test of the perovskite films. The TRPL data are fitted by a biexponential decay model and the corresponding lifetime values are listed in the inset of Figure 7b. The average carrier lifetime of TiO_2_/perovskite film is 110.5 ns. After the introduction of the ZrO_2_ insulating layer for TiO_2_, the average carrier lifetime reduces to 82.5 ns, indicating improved charge transport. However, for the TiO_2_/Al_2_O_3_/perovskite and TiO_2_/SiO_2_/perovskite films, the carrier lifetime increases to 125.6 and 108.3 ns, respectively.

To further investigate the kinetics of charge transport and recombination in perovskite solar cells, we measured the electrical impedance spectroscopy (EIS). The Nyquist plots of our C-PSCs, employing different insulating layers, are shown in Figure 7c. The semicircles at high- and low-frequency regions can be assigned to the charge transport resistance (R_ct_) and recombination resistance (R_rec_), respectively [43]. The corresponding impedance parameters are listed in Table 2. It is found that R_ct_ slightly decreases with the addition of ZrO_2_ or SiO_2_, indicating that the presence of ZrO_2_ or SiO_2_ has a slight promoting effect on charge transport. However, after Al_2_O_3_ treatment, R_ct_ increases from 44.5 to 50.4 Ω due to hindered charge transport by the addition of dense Al_2_O_3_. On the other hand, R_rec_ obviously increases with the incorporation of insulating layers, indicating effectively suppressed charge recombination, which confirms our previous expectation that insulating layers can prevent direct contact with carbon and TiO_2_ [38]. In general, ZrO_2_-based C-PSC has the smallest R_ct_ and the largest R_rec_, indicating faster carrier transport and slower recombination, which well explains the significant improvement in the V_oc_ and FF of corresponding devices. The buried interface quality has been highly improved due to carrier modulation with highly suppressed nonradiative recombination.

**Figure 8:**
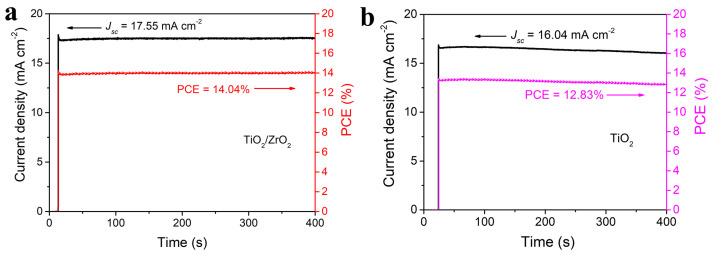
Steady-state photocurrent and PCE output as a function of time held at a bias of 0.80 V under one-sun (100 mW cm^−2^) illumination for the device based on (**a**) TiO_2_/ZrO_2_ and (**b**) pristine TiO_2_.

To further confirm the reliability of our fabricated C-PSCs, the steady-state efficiency of C-PSC fabricated on TiO_2_/ZrO_2_ is measured in ambient air under a constant bias of 0.8 V near the maximum power point. As shown in Figure 8a, our device presents a stable current density of 17.55 mA cm^−2^ under continuous illumination for 400 s, and the corresponding PCE is 14.04%. In comparison, the original TiO_2_-based device produces only 12.83% steady-state PCE under the same test condition, with a current density of 16.04 mA cm^−2^ (Figure 8b).

**Figure 9:**
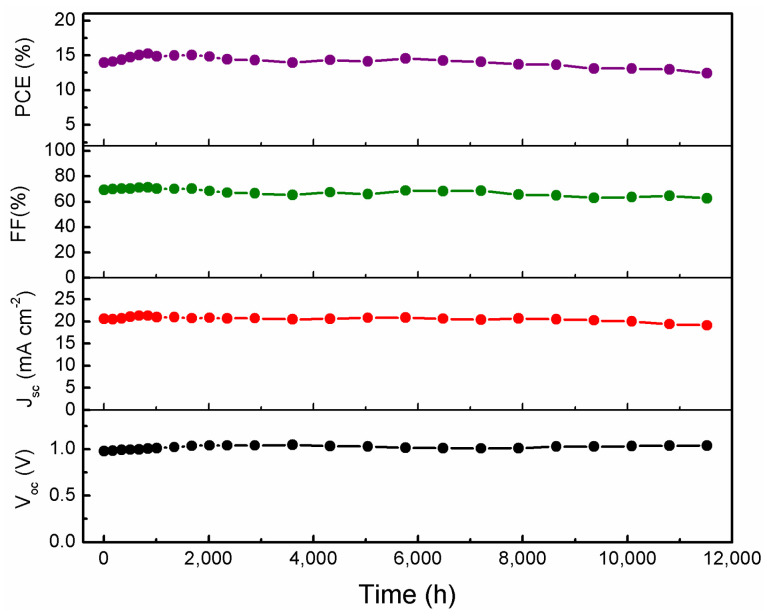
Photovoltaic parameters versus storage time for a TiO_2_/ZrO_2_/MAPbI_3_/carbon solar cell stored under dry air with humidity of 50% at room temperature without encapsulation.

Since the stability of PSC is one of the most critical concerns for the future commercialization of the devices, we have also recorded the stability of C-PSCs prepared on TiO_2_/ZrO_2_ to verify the long-term endurance in ambient air conditions with a temperature of 25 °C and humidity of 50 RH%. As shown in Figure 9, the V_oc_ slightly increases during the stability test, while J_sc_ and FF first increase and then show a decreasing trend. The PCE increases from an initial 13.96% to the highest of 15.24%, and finally drops to 12.41% after being stored for 11,520 h (480 days), demonstrating the outstanding stability of C-PSCs, which is among the first class of state-of-the-art devices [18,32]. The better performance during storage may be ascribed to the better contact attained between the perovskite layer and the carbon CE [43]. We further investigated the thermal stability of our unencapsulated devices by placing them on a heating plate at 85 °C in an environment with a humidity of 50%. Figure S3 shows the variation in PCE with heating time. The PCE initially improved slightly; however, it reduced to 81% of the initial value after 120 h of continuous heating. This may be caused by the decomposition of perovskite material in unencapsulated devices triggered by the high-humidity environment.

## 4. Conclusions

In summary, ZrO_2_, Al_2_O_3,_ and SiO_2_ are successfully used as insulating TOP layers for air-processed, highly efficient and stable C-PSCs. These common insulating materials can effectively separate TiO_2_ ETL and carbon electrodes, thus efficiently inhibiting carrier recombination caused by shunting. The main reason for the variation in improving the performance of C-PSCs lies in the morphology of insulating layers, which affects the infiltration and growth of perovskite material. We achieved the best performance of C-PSCs with a PCE of 14.96% using TiO_2_/ZrO_2_ as a scaffold layer, indicating that ZrO_2_ is the most suitable insulating layer for the system of C-PSCs. Moreover, our C-PSCs show outstanding long-term stability, maintaining 88.9% of their initial efficiency after 11,520 h storage in ambient air. This work is promising for high performance carbon-based HTM-free perovskite solar cells via the optimization of insulation materials. The high efficiency and stability in our TOP layer passivated C-PSCs offer a step towards the future commercialization of this low-cost photovoltaic technology.

## Figures and Tables

**Table 1:** Average photovoltaic parameters of total of 160 C-PSCs prepared with different scaffold layers. The error values represent the standard deviations.

Scaffold Layer	V_oc_ (V)	J_sc_ (mA/cm^2^)	FF (%)	PCE (%)
TiO_2_	0.959 ± 0.015	20.29 ± 0.42	65.25 ± 1.82	12.71 ± 0.45
TiO_2_/ZrO_2_	0.985 ± 0.018	20.61 ± 0.47	68.18 ± 1.63	13.84 ± 0.53
TiO_2_/Al_2_O_3_	0.969 ± 0.014	20.34 ± 0.54	65.39 ± 1.83	12.89 ± 0.56
TiO_2_/SiO_2_	0.971 ± 0.013	20.63 ± 0.56	65.39 ± 1.83	13.42 ± 0.48

**Table 2:** Impendence values of PSCs with different insulating layers.

Samples	R_s_ (Ω)	R_ct_ (Ω)	R_rec_ (Ω)
TiO_2_	52.0	44.6	126.5
TiO_2_/ZrO_2_	53.2	40.5	630
TiO_2_/Al_2_O_3_	57.1	50.4	185
TiO_2_/SiO_2_	55.3	42.1	280

## Data Availability

Not applicable.

## Supplementary Materials

The following supporting information can be downloaded at: https://www.mdpi.com/article/10.3390/nano13192640/s1, Figure S1: The N_2_ adsorption–desorption isotherms and corresponding pore size distribution curves (inset) of (a) TiO_2_, (b) ZrO_2_, (c) Al_2_O_3_ and (d) SiO_2_; Figure S2: Histograms of solar cell efficiencies were collected from 40 cells with ZrO_2_ TOP; Figure S3: Environmental thermal stress (85 °C in a heating panel) with high humidity (40–60%) conditions.
