# Supplementary material for: Carrier Modulation via Tunnel Oxide Passivating at Buried Perovskite Interface for Stable Carbon-Based Solar Cells
Source: Nanomaterials (Basel). 2023 Sep 26;13(19):2640. doi: 10.3390/nano13192640 (PMC10574625; doi:10.3390/nano13192640)
Supplement: Supplementary file 1 [file nanomaterials-13-02640-s001.zip › nanomaterials-2587567-supplementary.pdf]

Carrier Modulation via Tunnel Oxide Passivating at Buried Perovskite Interface for Stable Carbon-Based Solar Cells

Yuqing Xiao [1,2,*], Huijie Zhang [2], Yue Zhao [3], Pei Liu [2], Kiran Kumar Kondamareddy [4] and Changlei Wang [3,*]

1 School of Automation, Zhongkai University of Agriculture and Engineering, Guangzhou 510225, China
2 Key Laboratory of Artificial Micro & Nano Structures of Ministry of Education, School of Physics and Technology, Wuhan University, Wuhan 430072, China; zhanghj@whu.edu.cn (H.Z.); liup@whu.edu.cn (P.L.)
3 Key Laboratory of Advanced Optical Manufacturing Technologies of Jiangsu Province & Key Laboratory of Modern Optical Technologies of Education Ministry of China, School of Optoelectronic Science and Engineering & Collaborative Innovation Center of Suzhou Nano Science and Technology, Soochow University, Suzhou 215006, China; 20204039003@stu.suda.edu.cn
4 Department of Physics, School of Pure Sciences, College of Engineering Science and Technology,
FIJI National University, Lautoka Campus, Suva 744101, Fiji; kiran.kondamareddy@fnu.ac.fj
* Correspondence: xiaoyuqing@whu.edu.cn (Y.X.); cl.wang@suda.edu.cn (C.W.)

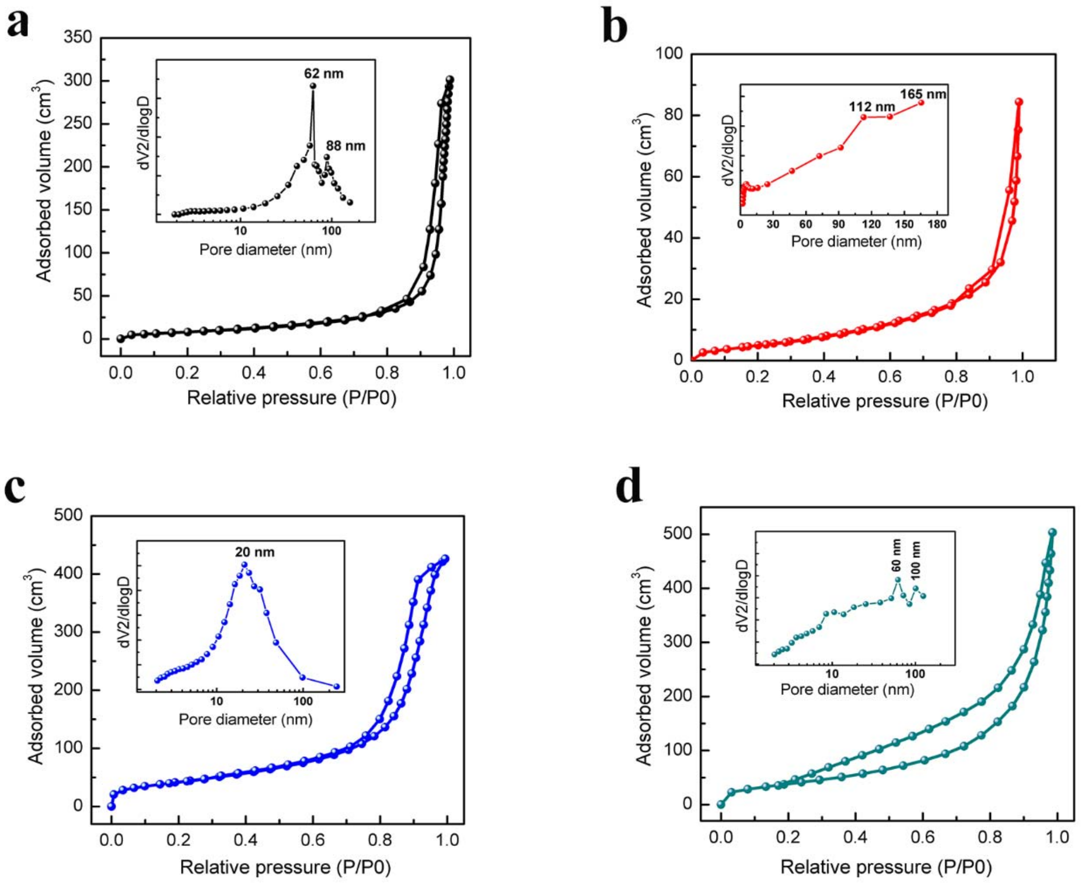

Figure S1. The N_2 adsorption-desorption isotherms and corresponding pore size

distribution curves (inset) of (a) TiO_2, (b) ZrO_2, (c) Al_2O_3, and (d) SiO_2.

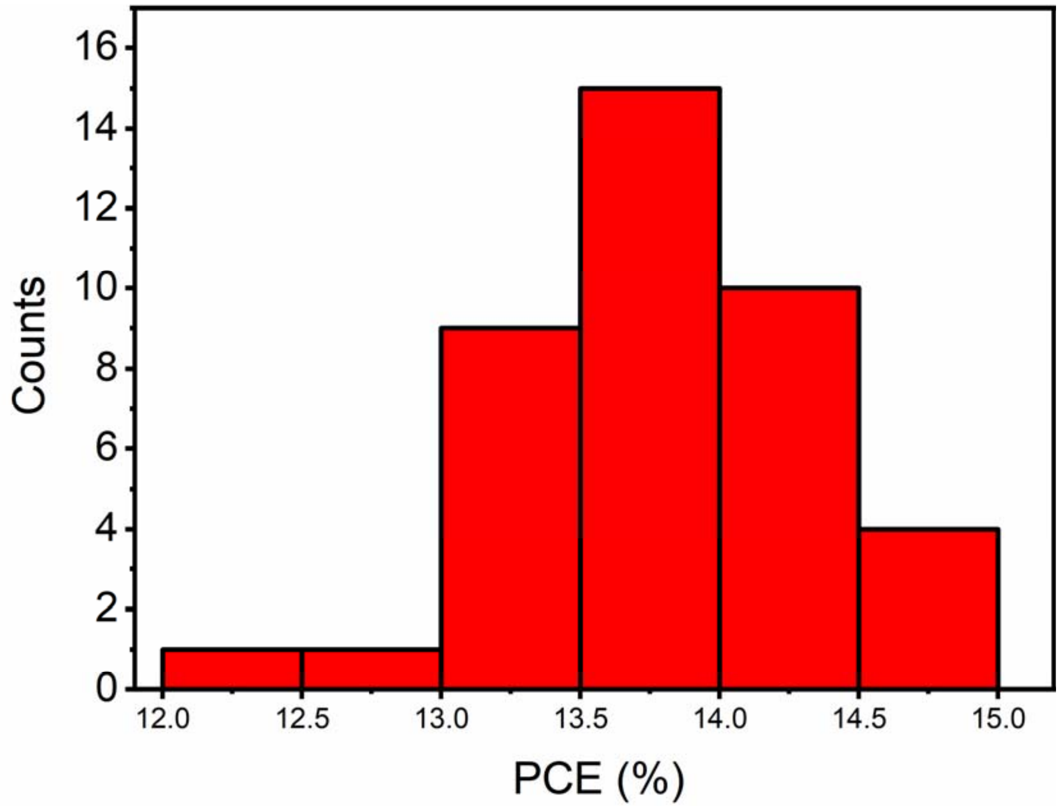

Figure S2. Histograms of solar cell efficiencies were collected from 40 cells with ZrO_2 TOP.

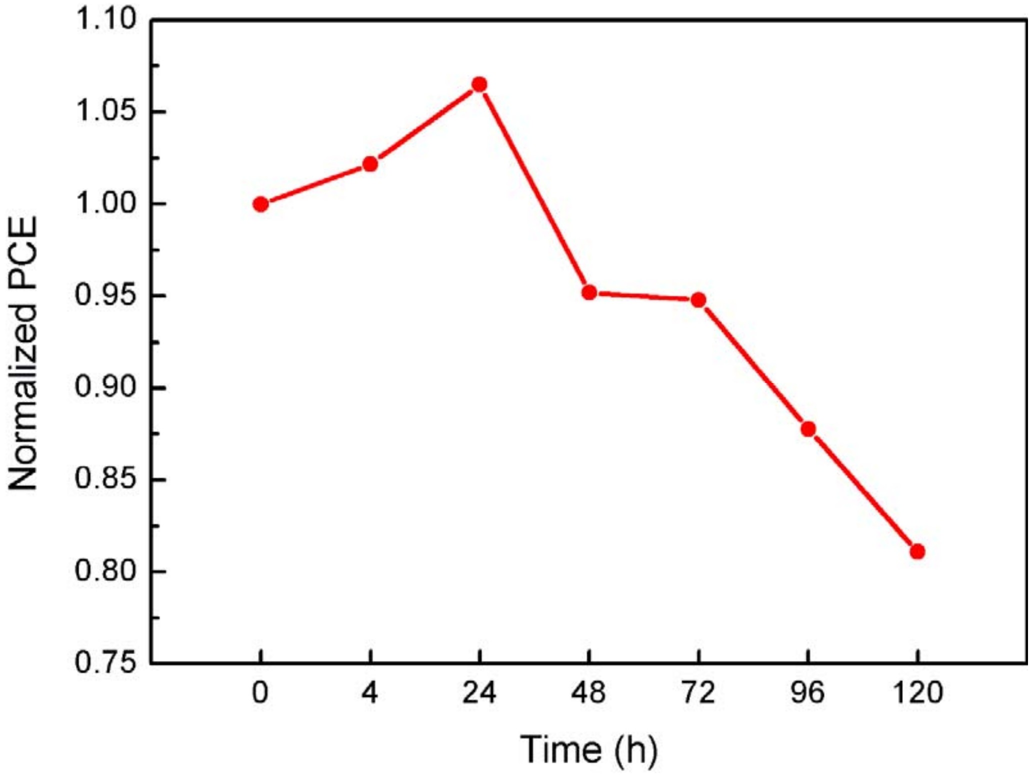

Figure S3. Environmental thermal stress (85 °C in a heating panel) with high humidity (40−60%) conditions.
